# Life satisfaction and motives for postponing parenthood among Polish women: A sequential indirect association model of depressive symptoms, future anxiety, and procrastination

**DOI:** 10.1371/journal.pone.0361199

**Published:** 2026-11-12

**Authors:** Małgorzata Szcześniak, Agata Hiacynta Świątek, Mariia Ivanytska, Adam Falewicz, Radosław Rybarski, Joanna Pracka, Rafał Pietruszka

**Affiliations:** 1 Institute of Psychology, University of Szczecin, Szczecin, Poland; 2 Institute of Psychology, John Paul II Catholic University of Lublin, Lublin, Poland; 3 Faculty of Social Sciences, The Pontifical University of John Paul II, Kraków, Poland; University of Padova, ITALY

## Abstract

Postponed parenthood is an increasingly common feature of reproductive patterns in high- and middle-income societies. Although previous research has examined factors associated with delayed parenthood, less is known about the psychological characteristics linked to self-reported motives for postponement. The present study examined the relationship between life satisfaction and motives for postponing parenthood and tested a theoretically ordered cross-sectional model involving depressive symptoms, future anxiety, and procrastination. Participants were 201 childless Polish women aged 20–44 years (*M* = 32.75, *SD* = 6.91). They completed the Satisfaction with Life Scale, the Multidimensional Scale of Motives for Postponing Parenthood, the Brief Screen for Depression, the Dark Future Scale, and the Pure Procrastination Scale. Higher life satisfaction was negatively related to some, but not all, motive-specific outcomes and to depressive symptoms. Depressive symptoms were positively associated with future anxiety, which was, in turn, positively related to procrastination. Higher procrastination was linked to higher motive-specific and overall postponement scores. The hypothesized specific sequential indirect association of life satisfaction with motives through depressive symptoms, future anxiety, and procrastination was statistically supported for uncertainty and incompetence (*B* = −.0083, 95% bootstrap CI [−.0214, − .0007]), fear of change (*B* = −.0054, 95% bootstrap CI [−.0148, − .0003]), financial security concern (*B* = −.0095, 95% bootstrap CI [−.0229, − .0012]), and the overall motives score (*B* = −.0324, 95% bootstrap CI [−.0818, − .0027]). The corresponding sequential indirect associations were not supported for self-focus, parenthood as a burden, or worry about a child’s future, indicating that the pattern differed across motives. Sensitivity analyses indicated that some outcome-specific sequential associations were not robust to the exclusion of potential multivariate outliers. Overall, the findings suggest that life satisfaction, depressive symptoms, future anxiety, and procrastination show distinct associations with self-reported reasons for postponing parenthood. Given the cross-sectional design, the observed sequential indirect associations should not be interpreted as evidence of temporal or causal psychological mechanisms.

## 1. Introduction

Postponed parenthood represents a major shift in contemporary reproductive patterns, particularly in high- and middle-income societies [1–4]. According to the Central Statistical Office in Poland [5], the average age of Polish women at the birth of their first child is approximately 29 years, which is close to the European Union average in 2022 (about 30 years). An increase in age at first childbirth has also been observed in North and South America [6,7], Asia [8], and Australia [9]. This broader pattern is further confirmed by population-level data: across OECD countries, the mean age of women at first birth increased from 26.4 years in 2000 to 29.5 years in 2022 [10], while Eurostat reports that the mean age of first-time mothers in the European Union reached 29.8 years in 2023 [11].

Delaying parenthood to the mid-30s or later appears to reflect an interplay of individual, relational, socioeconomic, and structural factors [12,13]. Mills et al. [13] identify changes in values, effective contraception, women’s participation in the labor market and higher education, economic uncertainty, partnership changes, family policies, and housing conditions as relevant factors. For example, among Australian men, fatherhood is more often preferred once a stable close relationship, personal maturity, completed education, and flexible but stable employment have been achieved [9]. These observations are consistent with broader OECD evidence indicating that fertility and family formation are related to economic and financial security, labor-market conditions, housing costs, and the family-policy environment [10].

The decision to have a child may therefore be preceded by the fulfillment of several perceived conditions [12] and may depend partly on expectations of a stable and predictable future. Periods of pronounced uncertainty, such as the COVID-19 pandemic, have been associated with revisions of fertility plans rather than with a uniform effect on reproductive decision-making [14–16]. In a five-country European study, fertility plans were negatively revised during the pandemic, with postponement observed in several countries [16]. Similarly, a Polish study found that pandemic-related concerns were associated with changes in reproductive intentions; among respondents who changed their plans, 74.1% intended to have a child later than previously planned [16]. More broadly, longitudinal population-level evidence from Germany indicates that several personal and social worries were associated with a lower likelihood of childbearing, supporting a cautious link between perceived uncertainty about the future and reproductive behavior [17]. Contemporary parenthood is also increasingly conceptualized as a consciously planned undertaking [18] and is often associated with intensive, child-centered parenting that requires substantial emotional, practical, and financial investment [19].

At the same time, medically assisted reproduction does not eliminate the biological consequences associated with delayed parenthood [20]. Misconceptions about fertility may contribute to involuntary childlessness among women who postpone motherhood for occupational or other reasons [21]. Sobotka [22, p. 58] suggests that ages 36–42 may be regarded as a “critical window for their last pregnancy attempts,” given the increase in infertility after age 35. Deferred parenthood may also create additional life-course challenges, such as an overlap between caring for young children and aging parents.

A deeper understanding of the psychological factors associated with motives for postponing parenthood may therefore be valuable both theoretically and for the development of supportive social policies. Previous research [1–4] identified six major motives for postponing parenthood: uncertainty about one’s parenting skills, perceiving parenthood as a burden, self-focus, financial concerns, fear of changes associated with having a child, and concerns about the child’s future. Despite the identification of these motives, the psychological characteristics accompanying stronger endorsement of particular reasons for postponement remain insufficiently understood.

The existing literature has largely examined psychological correlates of reproductive intentions and postponement through separate or pairwise associations. Studies using the MSMPP-18 have also investigated indirect associations between psychological characteristics and motives for postponing parenthood. Specifically, parental maturity has been examined as a mediator of the association between trait anxiety and motives for postponing parenthood [3], while generational time perspective and life satisfaction have been investigated as parallel mediators of the association between future anxiety and these motives [4]. Less is known, however, about whether these psychological characteristics can be organized into a theoretically ordered sequence of associations with motives for postponing parenthood. The present study addresses this gap by integrating life satisfaction, depressive symptoms, future anxiety, and procrastination within a single cross-sectional model of sequential indirect associations rather than treating these constructs solely as separate correlates.

Construal Level Theory (CLT) [23,24] provides a broader conceptual perspective for understanding motives for postponing parenthood. According to CLT, psychologically distant events tend to be represented more abstractly than events perceived as close in time or experience. Parenthood may be construed as a distant future possibility, particularly when its consideration is linked to the fulfillment of other conditions, such as establishing a stable relationship, completing education, achieving financial security, or reaching personal readiness [13,25]. From this perspective, motives for postponing parenthood may partly reflect the psychological positioning of parenthood as temporally distant or contingent on other life conditions. Importantly, endorsement of such motives does not in itself indicate that an individual has formed and subsequently delayed a specific plan for parenthood [23,24]. Research in other behavioral domains also suggests that psychological distance can influence how individuals think about and engage with future-oriented actions [26,27]. However, the present study does not directly assess psychological distance, temporal distance, or level of construal. Accordingly, CLT is used as a broader conceptual background for understanding psychological characteristics potentially associated with motives for postponing parenthood rather than as a theoretical model directly tested in the present study. The primary focus is the proposed sequence of associations among life satisfaction, depressive symptoms, future anxiety, procrastination, and motives for postponing parenthood.

Accordingly, the present study examines how these variables are organized within a theoretically ordered cross-sectional model of sequential indirect associations. The model begins with life satisfaction, defined as a general evaluation of contentment with one’s life as a whole and an important component of subjective well-being [28]. Evidence from developed societies suggests that life satisfaction is positively associated with reproductive behavior. Longitudinal research among Australian women and men showed that prior life satisfaction was positively related to subsequent fertility [29]. Similarly, Mencarini et al. [30] found that higher life satisfaction was associated with reproductive behavior across seven low-fertility countries, suggesting that individuals who are more satisfied with their lives may feel better prepared for the challenges of childrearing. Life satisfaction has also been associated with subsequent childbearing in other research [31]. Taken together, previous findings suggest that life satisfaction, depressive symptoms, future anxiety, and procrastination may be interrelated rather than functioning as independent correlates of motives for postponing parenthood. Lower life satisfaction may be associated with greater depressive symptoms and more pronounced future-related concerns, which may, in turn, coincide with a greater tendency to delay important life decisions. These relationships provide a rationale for examining the variables jointly within a cross-sectional model of sequential indirect associations with motives for postponing parenthood.

Feeling unsuccessful in important life domains may negatively affect life satisfaction [32]. Young adulthood is characterized by increasing autonomy and self-direction, but also by instability and uncertainty related to adult roles and developmental tasks [33]. Lower life satisfaction is associated with greater anxiety and depressive symptomatology in both clinical and non-clinical populations [34]. In the context of reproductive decision-making, lower satisfaction with one’s current life may therefore be associated with stronger endorsement of reasons for postponing parenthood. Thus, we hypothesized that:

H1: Higher life satisfaction will be negatively associated with motives for postponing parenthood.

A substantial body of research indicates an inverse association between life satisfaction and depressive symptoms across adolescent and adult populations [35–42]. Lower life satisfaction has been associated with poorer affective functioning and greater depressive symptomatology, whereas improvements in life satisfaction may co-occur with reductions in depressive symptoms [36,40]. These findings support positioning depressive symptoms as the first intermediate variable in the specified sequence. Accordingly, we hypothesized that:

H2: Higher life satisfaction will be negatively associated with depressive symptoms.

Within this sequence, depressive symptoms may be accompanied by a more negative cognitive orientation toward the future, providing a conceptual link to future anxiety. Thinking about the future in negative or threatening terms is related to both future-negative orientation and difficulty remaining focused on the present [43]. Psychological research indicates that anticipating adverse events is characteristic of depressive cognition and may reflect both reduced positive expectations and increased expectations of negative outcomes [44]. Beck’s cognitive account of depression emphasizes hopelessness, self-deprecation, and loss as features that permeate thinking about both the past and the future [45,46]. Research among adolescents and adults further demonstrates associations between depressive symptoms, diminished agency, and negative expectations concerning future events [47,48]. Experimental findings likewise indicate that depressed individuals generate fewer positive future events and engage in more negative future thinking [49,50]. Taken together, these findings support an association between depressive symptoms and anxiety concerning the future. Accordingly, we hypothesized that:

H3: Higher levels of depressive symptoms will be positively associated with future anxiety.

Within the proposed theoretical ordering, future anxiety may be relevant to whether difficult or consequential actions are approached or postponed. Research indicates that present fatalism and negative temporal orientation may be linked to procrastination [51,52]. Procrastination is also positively related to anxious and uncertain thinking about the future [53], while individuals who procrastinate tend to report higher levels of depression, anxiety, stress, and self-doubt [54,55]. Difficulty regulating unpleasant emotions, worry, and catastrophizing have likewise been associated with procrastinatory behavior [56]. These findings suggest that postponing important tasks may be related to anxiety, uncertainty about the consequences of one’s actions, and difficulties coping with unpleasant emotional states [57]. On this basis, we hypothesized that:

H4: Higher levels of future anxiety will be positively associated with procrastination.

When future-oriented decisions are experienced as uncertain, threatening, or emotionally demanding, postponement may be consistent with attempts to avoid aversive emotional states. This provides a theoretical rationale for examining generalized procrastination in relation to future anxiety and stronger endorsement of motives for postponing parenthood.

Procrastination, defined as the voluntary delay of intended tasks or goals, is common among young adults [58]. Although it has been studied primarily in academic and occupational contexts, it may also extend to developmental goals and major life decisions [59]. Parenthood can be regarded as one possible developmental transition involving adult roles and responsibilities [60], and delayed parenthood has occasionally been described as “procreative procrastination” [61]. However, this term may oversimplify reproductive decision-making because postponing parenthood can reflect rational constraints, the absence of a desire for children at an earlier age, lack of a partner, or the substantial sacrifices that parenthood would require. Nevertheless, examining generalized procrastination in relation to motives for postponing parenthood remains relevant because a broader tendency to delay difficult or consequential actions may also be related to stronger endorsement of reasons for postponement [62].

In the present study, procrastination was assessed as a general dispositional tendency rather than as parenthood-specific procrastination. It was conceptualized as an intermediate variable in the proposed sequence because future anxiety may be associated with a broader tendency to delay difficult, uncertain, or consequential actions. Procrastination is commonly conceptualized as a form of self-regulatory failure and may serve short-term mood regulation or avoidance of aversive actions [63,64]. Evidence from academic procrastination is therefore used only as indirect support for this broader self-regulatory and avoidance process, rather than as evidence that academic and reproductive decisions are equivalent. Decisions about parenthood are more complex, relational, and value-laden and are additionally shaped by partnership, health, economic, and broader social circumstances [13]. Any cross-domain generalization should consequently be interpreted cautiously. In this context, generalized procrastination may be associated with avoidance when thinking about the future is characterized by negativity or uncertainty. Thus, we hypothesized that:

H5: Higher levels of generalized procrastination will be positively associated with stronger motives for postponing parenthood.

The evidence reviewed above provides a basis for the proposed sequence of the study variables. Life satisfaction is inversely associated with depressive symptoms and anxiety [65–72], whereas anxiety and negative future orientation have been linked to procrastination [51–57,73–75]. Much of the existing literature, however, has examined these relationships separately and often in contexts unrelated to reproductive decision-making.

The proposed sequence reflects a progression from a broad evaluation of one’s current life circumstances to more specific affective, future-oriented, and behavioral characteristics. Life satisfaction was selected as the starting point because it represents a global cognitive evaluation of subjective well-being, whereas depressive symptoms capture a more specific form of negative affective functioning. Depressive symptoms precede future anxiety in the model because depressive cognition is commonly characterized by positive expectations, and negative representations of what lies ahead. Future anxiety, in turn, precedes procrastination because uncertainty or perceived threat regarding anticipated outcomes has been associated with avoidance and the postponement of difficult or consequential actions. Procrastination was therefore considered the construct conceptually most proximal to motives for postponing parenthood. This sequence should be understood as one theoretically plausible arrangement of the constructs rather than as an established temporal or causal pathway. Given the cross-sectional design, reciprocal, parallel, and partly reversed associations remain possible. The model therefore evaluates whether the observed associations are consistent with the proposed structure, not whether one variable temporally precedes or causally produces another.

Motives for postponing parenthood are heterogeneous rather than interchangeable. Some motives concern individual evaluations of readiness, burden, self-development, or anticipated lifestyle change, whereas others relate more strongly to contextual concerns such as finances or the future conditions in which a child would be raised [1–4]. The proposed sequence of associations may consequently not be equally evident across all motives. Examining both the total score and the six specific motives allows us to assess whether the sequential indirect association is relatively general or particularly relevant to certain reasons for postponement. The total MSMPP-18 score was therefore treated as the primary outcome representing the overall level of motives for postponing parenthood, whereas the six motive-specific dimensions were examined as secondary outcomes to characterize possible heterogeneity in the observed associations. This motive-specific examination constitutes a secondary contribution of the present study.

Against this background, the central contribution of the present study is the examination of an integrated cross-sectional model of sequential indirect associations linking life satisfaction with motives for postponing parenthood through depressive symptoms, future anxiety, and procrastination (Figure 1). The individual hypotheses specify the component associations within this broader model, whereas the sequential indirect association constitutes its primary theoretical test.

**Fig 1:**
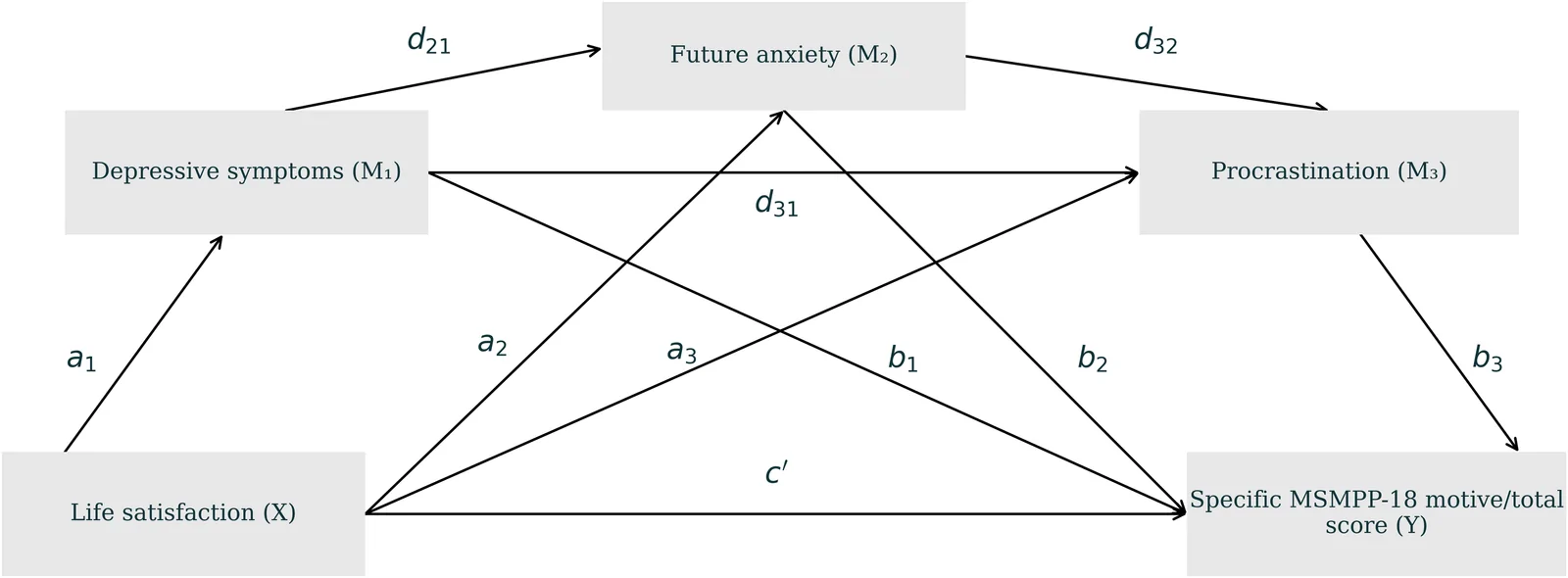
Conceptual sequential indirect-association model linking life satisfaction with motives for postponing parenthood through depressive symptoms, future anxiety, and procrastination. Note. Paths *a*_1_–*a*_3_ indicate associations between life satisfaction and intermediate variables; *d*_21_, *d*_31_, and *d*_32_ indicate associations among the mediators; *b*_1_–*b*_3_ indicate associations between the mediators and the outcome; and c′ denotes the direct association between life satisfaction and the outcome. The total association (c) was estimated separately without the mediators. The model was estimated for each of the six MSMPP-18 motives and for the total score.

H6: Life satisfaction is expected to show a statistically significant sequential indirect association with motives for postponing parenthood through depressive symptoms, future anxiety, and procrastination.

## 2. Methods

### 2.1. Participants and procedure

A total of 201 Polish women participated in the study. The age range of the sample was 20–44 years (*M* = 32.75; *SD* = 6.91). With respect to place of residence, 23.9% of the women were from villages; 23.4% were from cities with between 100,000 and 500,000 inhabitants; 20.9% were from towns with between 10,000 and 50,000 inhabitants; 19.4% were from cities with more than 500,000 inhabitants; and 12.4% were from cities with between 50,000 and 100,000 inhabitants. In terms of education, most women reported having higher education (51.2%), followed by secondary education (38.8%), vocational education (7.0%), and primary education (3.0%). Regarding relationship status, most participants reported being in a partnership (42.3%), followed by being single (28.9%), married (19.4%), engaged (5.4%), and divorced (4.0%).

Participants were recruited between March and April 2025 through social media platforms, including Facebook and Instagram, using a convenience sampling strategy. Recruitment posts invited women without children to participate in an online survey. Eligible respondents were Polish women aged 18–45 years who had no children. Eligibility was assessed at the beginning of the survey through self-report questions concerning nationality, age, and parental status. Taking part was voluntary, and no financial or other incentives were offered. Thus, the sample consisted of childless women who completed a measure assessing motives for postponing parenthood; childlessness itself was not treated as evidence that participants had actually postponed parenthood or intended to become parents in the future. The study was limited to women because the timing of parenthood is subject to sex-specific biological reproductive constraints, which were directly relevant to the age criterion adopted in the present study. The upper age limit of 45 years was adopted as an operational criterion reflecting the marked age-related decline in female reproductive potential. Evidence from natural-fertility populations indicates that nearly 90% of women have reached the end of natural fertility by this age [76]. Moreover, women aged 45 years and older are classified in the literature as being of very advanced maternal age [77]. A total of 201 participants started and completed the survey. No participants were excluded because all respondents met the eligibility criteria. There were no missing data because the online survey required participants to provide a response to each item before proceeding to the next question. The present sample was entirely independent of the samples used in References 3 and 4.

All participants provided electronic informed consent at the beginning of the online survey after receiving information about the study purpose, minimal-risk nature, confidentiality, and their right to withdraw at any time without negative consequences. The study project was approved by the Research Ethics Committee of the Institute of Psychology at the University of Szczecin (No. 24/2023, November 9, 2023) and was conducted in accordance with the Declaration of Helsinki. The study was retrospectively registered on the Open Science Framework on April 10, 2026 (https://doi.org/10.17605/OSF.IO/EWG5N), after the data had been accessed and after at least some analyses had been conducted. Thus, this registration is not a preregistration and does not distinguish confirmatory from exploratory analytic decisions. Its purpose is to document the analytic procedures and promote analytic transparency. De-identified data and a variable codebook are available in the OSF repository at https://osf.io/ba7j6.

### 2.2. Measures

#### 2.2.1. Satisfaction with Life Scale.

The **Satisfaction with Life Scale** [SWLS], designed by Diener et al. [28] and adapted into Polish by Juczyński [78], is a five-item measure to assess global life satisfaction defined as the cognitive component of subjective well-being (e.g., “So far I have gotten the important things I want in life”). Each statement is rated on a 7-point scale, where 1 means *strongly disagree* and 7 means *strongly agree*. The total SWLS score ranges from 5 (the lowest life satisfaction) to 35 (the highest life satisfaction). The SWLS is a highly reliable scale with Cronbach’s alpha α = 0.88 [28]. In the current study, the internal consistency was also very high, with α = 0.92.

#### 2.2.2. Multidimensional Scale of Motives for Postponing Parenthood.

The **Multidimensional Scale of Motives for Postponing Parenthood** [MSMPP-18] developed by Szcześniak et al. [1] is a tool to measure six motives for delaying the decision about becoming a parent: (1) *feeling of uncertainty and incompetence* refers to the personal conviction about not being able to meet parental demands (e.g., “I do not have the required qualities to be a good enough parent”); (2) *self-focus* involves the tendency to emphasize self-fulfillment before embarking on parenthood (e.g., “Currently, achieving my aspirations is my priority”); (3) *parenthood as a burden* reflects a fear of sacrifices related to being a parent (e.g., “Parenting takes time and commitment”); (4) *fear of change* means the fear of negative alterations resulting from the birth of a child (e.g., “I am afraid of deterioration of sexual satisfaction in the relationship”); (5) *financial security* concern is associated with the belief that having a child is beyond one’s economic resources (e.g., “I do not have sufficient financial resources”); (6) *worry about a child’s future* indicates the fear that the child could live in unstable times and be exposed to climate change or wars (e.g., “I do not want my child to experience a climate catastrophe”). The MSMPP-18 not only measures six motives (each factor consisting of 3 items) but can also be treated as a single-factor tool on delayed parenthood. Study participants provide answers on a seven-point Likert scale (1 = *strongly disagree*, 7 = *strongly agree*). Previous psychometric validation of the MSMPP-18, including confirmatory factor analyses, supported its multidimensional structure and the use of an overall score reflecting the general level of motives for postponing parenthood [1,2]. Thus, the instrument can be interpreted both at the level of its six specific subscales and as a total score, depending on the purpose of the analysis. In previous studies, the internal consistency of the MSMPP-18 subscales ranged from acceptable to excellent (Cronbach’s α = .69–.95), although the lowest coefficient reflected only marginal reliability. In the present study, the results of Cronbach’s alpha were as follows: (1) feeling of uncertainty and incompetence (α = 0.91); (2) self-focus (α = 0.95); (3) parenthood as a burden (α = 0.71); (4) fear of change (α = 0.82); (5) financial security concern (α = 0.94); (6) worry about a child’s future (α = 0.81); and the total score of the MSMPP-18 (α = 0.93).

#### 2.2.3. Brief Screen for Depression.

The **Brief Screen for Depression** [BSD], designed by Hakstian and McLean [79] and translated into Polish by Zarzycka et al. [80], is a four-item scale for the initial assessment of depressive symptoms among non-clinical individuals. Scores above 21 have been suggested as indicating elevated depressive symptomatology rather than a clinical diagnosis. In the present sample, 58 participants (28.9%) scored above this threshold. Previous studies have shown acceptable internal reliability of the BSD (α = 0.65). In the current study, Cronbach’s alpha was satisfactory, with α = 0.79.

#### 2.2.4. Dark Future Scale.

The **Dark Future Scale** [DFS], developed by Zaleski et al. [43], is a five-item unidimensional scale that measures future anxiety that refers to a person’s attitude toward distant and potentially unfavorable changes in the future perspective (e.g., “I am terrified by the thought that I might sometimes face life’s crises or difficulties”). Participants indicate their attitude toward the future on a seven-point Likert scale, where 0 means *decidedly false* and 6 means *decidedly true*. Previous studies have confirmed that the DFS, although brief, has good psychometric properties with Cronbach’s α = 0.79. In the present study, the scale presented very good internal reliability, with α = 0.93.

#### 2.2.5. Pure procrastination scale.

The **Pure Procrastination Scale** [PPS], developed by Steel [81] and adapted into Polish by Stępień and Cieciuch [82], with subsequent modifications by Stępień and Topolewska [83], is a 12-item tool that measures voluntary delaying of an intended course of action even though a worse situation can be expected because of the delay (e.g., “I am not very good at meeting deadlines”). The statements are scored on a 5-point Likert scale (1 =  *very seldom or not at all like me*, 5  =  *very often or very true of me*). Although the PPS measures three types of procrastination (decisional delay, implemental delay, and timeliness-lateness), the overall score was used in the current study. The PPS has very good reliability both for its three dimensions (between α = 0.83 and α = 0.87) and for its total score (α = 0.92). In the present study, the internal consistency was also very good (α =  0.95).

### 2.3. Sample size and sensitivity analysis

To estimate the minimum required sample size a priori, a power analysis was conducted using G*Power 3.1.9.4 for a bivariate correlation [84,85]. Given the directional nature of the hypotheses, a one-tailed test was specified with α = .05, power = .95, and an effect size of *r* = .252, based on the average magnitude of previously reported associations between life satisfaction and postponement of parenthood [1–4]. The analysis indicated a minimum required sample size of *N* = 161. Because this calculation was based on a bivariate correlation, it did not establish statistical power for the more complex three-mediator sequential indirect effect. Therefore, a post hoc Monte Carlo sensitivity analysis was additionally conducted to evaluate the ability of the present sample to detect the hypothesized sequential indirect association.

### 2.4. Statistical analysis

Statistical analyses were performed using IBM SPSS Statistics (version 25). Measures of skewness and kurtosis were used to assess the distributions of the study variables. Although the Shapiro–Wilk tests were statistically significant, skewness and kurtosis values remained within ±2 (Table 1), indicating no substantial departures from normality. Pearson’s *r* correlations were calculated to examine associations among life satisfaction, depressive symptoms, future anxiety, procrastination, and the specific and overall scores for motives for postponing parenthood. For the Pearson correlation analyses, linearity was assessed by visual inspection of bivariate scatterplots.

**Table 1:** Descriptive statistics for life satisfaction, depressive symptoms, future anxiety, procrastination, motives for postponing parenthood and its overall score (*N* = 201).

	*M*	*SD*	Skewness	Kurtosis	Shapiro–Wilk	*p*
1. SAT	18.88	7.48	0.053	−0.352	0.976	0.002
2. DEP	17.93	7.61	−0.034	−0.449	0.974	0.001
3. FAN	16.89	7.80	−0.345	−0.245	0.960	0.001
4. PRO	33.43	12.53	0.110	−0.617	0.974	0.001
5. UNC	11.60	5.42	0.051	−1.014	0.955	0.001
6. SFO	13.37	5.20	−0.219	−0.822	0.953	0.001
7. BUR	15.22	4.14	−0.652	0.043	0.950	0.001
8. CHA	12.42	4.66	−0.205	−0.523	0.971	0.001
9. FIN	13.62	5.90	−0.295	−1.146	0.916	0.001
10. WOR	14.24	4.74	−0.514	−0.421	0.952	0.001
11. MPP	80.50	22.54	−0.286	−0.431	0.984	0.025

*Note*. SAT–Life satisfaction; DEP–Depressive symptoms; FAN–Future anxiety; PRO–Procrastination; UNC–Feeling of uncertainty and incompetence; SFO–Self-focus; BUR–Parenthood as a burden; CHA–Fear of change; FIN–Financial security concern; WOR–Worry about a child’s future; MPP–Total score for motives for postponing parenthood.

A hierarchical multiple regression analysis was conducted to examine the associations of relevant sociodemographic covariates with the total score for motives for postponing parenthood and to determine the additional variance associated with life satisfaction, depressive symptoms, future anxiety, and procrastination. Regression assumptions were evaluated through visual inspection of standardized residuals plotted against standardized predicted values for evidence of nonlinearity and heteroscedasticity, together with the distribution of residuals. Multicollinearity was assessed using tolerance and variance inflation factor (VIF) values, with tolerance values ≤ .10 and VIF values > 10 considered indicative of problematic multicollinearity. Potential multivariate outliers were identified using Mahalanobis distance with a criterion of *p* < .001, whereas Cook’s distance values > 1 were considered indicative of potentially influential observations.

Age and relationship status were selected as covariates on theoretical grounds because both are directly relevant to reproductive decision-making and the context in which motives for postponing parenthood are evaluated [1–4,86,87]. Age was entered as a continuous variable, whereas relationship status was binary-coded as 0 = not currently in a relationship (single, separated, divorced, or widowed) and 1 = currently in a relationship (partnered, engaged, or married). These covariates were specified a priori rather than based on statistical significance in preliminary analyses. Place of residence and education were reported descriptively but were not included as covariates. In the hierarchical regression analysis, age and relationship status were entered in Step 1, followed by life satisfaction, depressive symptoms, future anxiety, and procrastination in Step 2.

Seven cross-sectional models of sequential indirect associations were estimated using PROCESS Model 6 [88]. Life satisfaction (SAT) was entered as the focal predictor; depressive symptoms (DEP; M1), future anxiety (FAN; M2), and procrastination (PRO; M3) were entered as sequential intermediate variables; and the six specific motives for postponing parenthood and their overall score were examined as separate outcome variables. Age and relationship status were included as covariates in all intermediate-variable and outcome equations. Indirect associations were evaluated using 5,000 bootstrap resamples with 95% confidence intervals (CIs).

The seven outcome variables were: feeling of uncertainty and incompetence (UNC), self-focus (SFO), parenthood as a burden (BUR), fear of change (CHA), financial security concern (FIN), worry about a child’s future (WOR), and the total score for motives for postponing parenthood (MPP). Accordingly, seven cross-sectional models of sequential indirect associations were specified: SAT → DEP → FAN → PRO → UNC; SAT → DEP → FAN → PRO → SFO; SAT → DEP → FAN → PRO → BUR; SAT → DEP → FAN → PRO → CHA; SAT → DEP → FAN → PRO → FIN; SAT → DEP → FAN → PRO → WOR; and SAT → DEP → FAN → PRO → MPP. Specific indirect effects were considered statistically supported when their bootstrap 95% confidence intervals did not include zero.

Because the primary analyses involved relatively complex cross-sectional models of sequential indirect associations, an additional simulation-based Monte Carlo sensitivity analysis was conducted for the hypothesized specific sequential indirect association through depressive symptoms, future anxiety, and procrastination (*a*_1_*d*_21_*d*_32_*b*_3_). Path estimates and residual variances from the covariate-adjusted models were treated as plausible population parameters. The analysis evaluated the sensitivity of the obtained sample size (*N* = 201) to detecting this specific sequential indirect association across the seven outcomes.

## 3. Results

### 3.1. Descriptive statistics

Descriptive statistics for all study variables are presented in Table 1. Although the Shapiro–Wilk tests were significant, skewness and kurtosis values remained within ±2; therefore, Pearson’s correlations were used.

### 3.2. Multicollinearity, outliers, and confounders

Visual inspection of bivariate scatterplots revealed no marked departures from linearity among the variables included in the correlation analyses. Diagnostic checks for the hierarchical regression indicated no evidence of problematic multicollinearity, with VIF values ranging from 1.030 to 2.144 and tolerance values ranging from .467 to .971. Plots of standardized residuals against standardized predicted values showed no clear systematic pattern or substantial departures from linearity or homoscedasticity. The distribution of standardized residuals was approximately symmetric and showed no major deviations likely to materially affect the ordinary least squares (OLS) estimates. Three observations exceeded the critical Mahalanobis-distance value of 22.46 corresponding to χ^2^(6), *p* < .001, with distances of 24.582, 24.366, and 23.173. However, Cook’s distance values ranged from approximately 0 to .078 and were all well below 1, suggesting that none exerted undue influence on the regression estimates. These observations were therefore retained in the primary analyses.

A hierarchical multiple regression analysis was conducted with the total score for motives for postponing parenthood (MPP) as the outcome. Age and relationship status were entered in Step 1 and explained 9.9% of the variance, *R*^2^ = .099, adjusted *R*^2^ = .090, *F*(2, 198) = 10.881, *p* < .001. Age was significantly negatively associated with the MSMPP–18 score (*B* = −0.903, *SE* = 0.221, β = −.277, *p* < .001), whereas relationship status was not statistically significant (*B* = −6.114, *SE* = 3.241, β = −.128, *p* = .061).

Life satisfaction, depressive symptoms, future anxiety, and procrastination were entered in Step 2. The final model explained 34.4% of the variance in the total MSMPP–18 score, *R*^2^ = .344, adjusted *R*^2^ = .323, *F*(6, 194) = 16.936, *p* < .001, representing an additional 24.5% beyond age and relationship status, Δ*R*^2^ = .245, Δ*F*(4, 194) = 18.086, *p* < .001. Future anxiety (*B* = 0.671, *SE* = 0.223, β = .232, *p* = .003) and procrastination (*B* = 0.622, *SE* = 0.147, β = .346, *p* < .001) were positively associated with the total MSMPP–18 score, while age remained significantly negatively associated with the outcome (*B* = −0.679, *SE* = 0.192, β = −.208, *p* < .001). Relationship status (*B* = −2.302, *SE* = 2.906, β = −.048, *p* = .429), life satisfaction (*B* = −0.244, *SE* = 0.185, β = −.081, *p* = .190), and depressive symptoms (*B* = −0.091, *SE* = 0.252, β = −.031, *p* = .718) were not significant after adjustment for the remaining variables.

A sensitivity analysis excluding the three potential multivariate outliers produced substantively similar regression results. The model remained statistically significant, *R*^2^ = .334, adjusted *R*^2^ = .313, *F*(6, 191) = 15.946, *p* < .001, with no changes in the direction or statistical interpretation of the predictors. The conclusions of the hierarchical regression were therefore robust to the exclusion of these observations.

### 3.3. Correlations

Table 2 presents Pearson correlations and 95% confidence intervals among the study variables. H1 was partially supported: life satisfaction was negatively associated with uncertainty and incompetence, fear of change, financial security concern, and the overall motives score, but not with self-focus, parenthood as a burden, or worry about a child’s future. Consistent with H2, life satisfaction was negatively associated with depressive symptoms. Depressive symptoms were positively associated with future anxiety (H3), and future anxiety was positively associated with procrastination (H4). Procrastination was positively associated with all six motives for postponing parenthood and with the overall motives score, supporting H5.

**Table 2:** Correlations of the study variables (N = 201).

	SAT	DEP	FAN	PRO	UNC	SFO	BUR	CHA	FIN	WOR	MPP
SAT	1										
DEP	−0.21** [−.34; −.07]	1									
FAN	−0.21** [−.34; −.07]	0.59*** [.49; .67]	1								
PRO	−0.03 [−0.17;0.11]	0.65*** [0.56;0.72]	0.56*** [0.45;0.64]	1							
UNC	−0.18* [−0.31;−0.04]	0.40*** [0.27;0.51]	0.39*** [0.27;0.50]	0.49*** [0.37;0.58]	1						
SFO	0.13 [−0.01;0.26]	0.22** [0.08;0.35]	0.16* [0.02;0.29]	0.27*** [0.14;0.40]	0.32*** [0.19;0.44]	1					
BUR	−0.12 [−0.25;0.02]	0.17* [0.03;0.30]	0.32*** [0.19;0.44]	0.28*** [0.15;0.40]	0.53*** [0.42;0.62]	0.39*** [0.26;0.50]	1				
CHA	−0.15* [−0.28;−0.01]	0.33*** [0.20;0.45]	0.44*** [0.32;0.55]	0.42*** [0.29;0.52]	0.66*** [0.57;0.73]	0.40*** [0.27;0.51]	0.62*** [0.52;0.70]	1			
FIN	−0.28*** [−0.40;−0.14]	0.37*** [0.25;0.49]	0.42*** [0.30;0.53]	0.48*** [0.37;0.58]	0.66*** [0.58;0.73]	0.39*** [0.26;0.50]	0.50*** [0.39;0.60]	0.61*** [0.52;0.69]	1		
WOR	−0.08 [−0.22;0.06]	0.14* [0.01;0.28]	0.31*** [0.18;0.43]	0.22** [0.08;0.34]	0.42*** [0.30;0.53]	0.29*** [0.16;0.41]	0.48*** [0.37;0.58]	0.47*** [0.35;0.57]	0.36*** [0.23;0.47]	1	
MPP	−0.16* [−0.29;−0.02]	0.37*** [0.25;0.49]	0.46*** [0.34;0.56]	0.49*** [0.38;0.59]	0.81*** [0.76;0.85]	0.62*** [0.53;0.70]	0.76*** [0.70;0.81]	0.83*** [0.78;0.87]	0.81*** [0.75;0.85]	0.66*** [0.57;0.73]	1

*Note*. * *p* < 0.05; ** *p* < 0.01; *** *p* < 0.001; SAT−Life satisfaction; DEP−Depressive symptoms; FAN−Future anxiety; PRO−Procrastination; UNC − Feeling of uncertainty and incompetence; SFO−Self-focus; BUR−Parenthood as a burden; CHA − Fear of change; FIN − Financial security concern; WOR−Worry about a child’s future; MPP − Total score for motives for postponing parenthood.

### 3.4. Sequential indirect associations of depressive symptoms, future anxiety, and procrastination

Seven cross-sectional models of sequential indirect associations were tested using PROCESS Model 6 to examine H6. Life satisfaction (SAT) was entered as the predictor, depressive symptoms (DEP), future anxiety (FAN), and procrastination (PRO) specified as sequential intermediate variables. The total MSMPP-18 score served as the primary outcome, while the six motive-specific subscale scores were examined as secondary outcomes. Indirect effects were estimated using 5,000 bootstrap samples and 95% percentile bootstrap confidence intervals.

Table 3 presents the results of the regression equations for the three intermediate variables, adjusted for age and relationship status. Higher life satisfaction was associated with lower depressive symptoms, whereas depressive symptoms were positively associated with future anxiety. Life satisfaction was not uniquely associated with future anxiety after adjustment for depressive symptoms and the covariates. In the procrastination equation, life satisfaction, depressive symptoms, and future anxiety were all positively associated with procrastination. Among the covariates, relationship status was significantly associated only with future anxiety, while age was not significantly associated with any of the intermediate variables. All three intermediate-variable equations were statistically significant, with the greatest proportion of variance explained for procrastination, followed by future anxiety, and the smallest proportion explained for depressive symptoms.

**Table 3:** Regression paths and covariate effects defining the common cross-sectional sequence of indirect associations from life satisfaction through depressive symptoms, future anxiety, and procrastination (*N* = 201).

Outcome equation	Path	Relationship	*B*	*SE*	*β*	*t*	*p*	95% CI
DEP	*a*_1_	SAT → DEP	−.2128	.0711	−.2089	−2.9928	.0031	[−.3530, −.0726]
DEP	−	Age → DEP	−.1246	.0766	−.1130	−1.6255	.1056	[−.2757, .0266]
DEP	−	Relationship status → DEP	.9248	1.1302	.0571	.8182	.4142	[−1.3041, 3.1537]
DEP model fit		*R*² = .0593; *F*(3, 197) = 4.1380; *p* = .0072						
FAN	*a*_2_	SAT → FAN	−.0685	.0604	−.0657	−1.1344	.2580	[−.1877, .0506]
FAN	*d*_21_	DEP → FAN	.5858	.0592	.5718	9.8913	<.001	[.4690, .7026]
FAN	−	Age → FAN	−.0310	.0641	−.0275	−.4840	.6289	[−.1575, .0954]
FAN	−	Relationship status → FAN	−2.9885	.9411	−.1802	−3.1755	.0017	[−4.8445, −1.1325]
FAN model fit		*R*² = .3838; *F*(4, 196) = 30.5161; *p* < .001						
PRO	*a*_3_	SAT → PRO	.2473	.0883	.1476	2.8017	.0056	[.0732, .4215]
PRO	*d*_31_	DEP → PRO	.8663	.1056	.5267	8.2033	<.001	[.6580, 1.0745]
PRO	*d*_32_	FAN → PRO	.4176	.1040	.2601	4.0149	<.001	[.2125, .6228]
PRO	−	Age → PRO	−.0727	.0934	−.0401	−.7779	.4376	[−.2569, .1116]
PRO	−	Relationship status → PRO	−2.0402	1.4053	−.0766	−1.4518	.1482	[−4.8118, .7313]
PRO model fit		*R*² = .4956; *F*(5, 195) = 38.3239; *p* < .001						

*Note*. SAT–life satisfaction; DEP–depressive symptoms; FAN–future anxiety; PRO–procrastination; *B*–unstandardized regression coefficient; *SE*–standard error; *β*–standardized regression coefficient; CI–confidence interval. Age and relationship status (0 = not currently in a relationship and 1 = currently in a relationship) were included as covariates. Paths *a*_1_–*a*_3_ represent the adjusted associations of SAT with the three intermediate variables; *d*_21_, *d*_31_, and *d*_32_ represent the adjusted associations among the intermediate variables. The 95% CIs refer to the unstandardized coefficients. These coefficients were identical across all seven outcome models because the focal predictor, intermediate-variable sequence, and covariates were held constant.

Table 4 shows that outcome-specific associations varied across motives for postponing parenthood. Procrastination was positively associated with uncertainty and incompetence, parenthood as a burden, fear of change, financial security concern, and the overall motives score. Future anxiety was positively related to parenthood as a burden, fear of change, worry about a child’s future, and the overall motives score. Depressive symptoms showed no significant unique associations with any outcome after adjustment for the other variables. Life satisfaction showed significant total associations with uncertainty and incompetence, self-focus, fear of change, and financial security concern, whereas significant direct associations remained only for self-focus and financial security concern. Age was negatively associated with several outcomes, while relationship status was significantly associated only with self-focus. Overall, these findings demonstrate substantial variation in the correlates of different motives for postponing parenthood.

**Table 4:** Outcome-specific regression paths, covariate associations, and total and direct associations of life satisfaction in the adjusted cross-sectional models of sequential indirect associations (*N* = 201).

Outcome	Path	Relationship	*B*	*SE*	*β*	*t*	*p*	95% CI
UNC	*b*_1_	DEP → UNC	.0387	.0635	.0543	.6096	.5428	[−.0865, .1640]
	*b*_2_	FAN → UNC	.0818	.0561	.1176	1.4579	.1465	[−.0289, .1925]
	*b*_3_	PRO → UNC	.1601	.0371	.3695	4.3107	< .001	[.0868, .2333]
	*c′*	SAT → UNC	−.0907	.0467	−.1250	−1.9428	.0535	[−.1828, .0014]
	−	Age → UNC	−.0496	.0485	−.0632	−1.0229	.3076	[−.1453, .0461]
	−	Relationship status → UNC	−.2781	.7326	−.0241	−.3796	.7047	[−1.7229, 1.1668]
	*c*	SAT → UNC	−.1176	.0508	−.1620	−2.3152	.0216	[−.2177, −.0174]
SFO	*b*_1_	DEP → SFO	.1069	.0644	.1566	1.6595	.0986	[−.0202, .2340]
	*b*_2_	FAN → SFO	−.0195	.0569	−.0293	−.3429	.7321	[−.1318, .0928]
	*b*_3_	PRO → SFO	.0599	.0377	.1443	1.5895	.1136	[−.0144, .1342]
	*c′*	SAT → SFO	.1390	.0474	.1999	2.9345	.0037	[.0456, .2325]
	−	Age → SFO	−.1856	.0492	−.2465	−3.7687	< .001	[−.2827, −.0884]
	−	Relationship status → SFO	−1.9076	.7434	−.1726	−2.5662	.0110	[−3.3738, −.4415]
	*c*	SAT → SFO	.1190	.0467	.1711	2.5495	.0115	[.0269, .2110]
BUR	*b*_1_	DEP → BUR	−.0829	.0524	−.1524	−1.5825	.1152	[−.1862, .0204]
	*b*_2_	FAN → BUR	.1317	.0463	.2479	2.8439	.0049	[.0403, .2230]
	*b*_3_	PRO → BUR	.0706	.0306	.2134	2.3047	.0222	[.0102, .1310]
	*c′*	SAT → BUR	−.0418	.0385	−.0754	−1.0855	.2791	[−.1178, .0342]
	−	Age → BUR	−.0905	.0400	−.1508	−2.2600	.0249	[−.1694, −.0115]
	−	Relationship status → BUR	−.4744	.6043	−.0539	−.7851	.4334	[−1.6663, .7174]
	*c*	SAT → BUR	−.0508	.0386	−.0917	−1.3183	.1889	[−.1269, .0252]
CHA	*b*_1_	DEP → CHA	−.0468	.0548	−.0764	−.8541	.3941	[−.1548, .0612]
	*b*_2_	FAN → CHA	.1876	.0484	.3138	3.8759	< .001	[.0921, .2830]
	*b*_3_	PRO → CHA	.1035	.0320	.2781	3.2336	.0014	[.0404, .1667]
	*c′*	SAT → CHA	−.0581	.0403	−.0932	−1.4437	.1504	[−.1375, .0213]
	−	Age → CHA	−.1038	.0418	−.1537	−2.4800	.0140	[−.1863, −.0212]
	−	Relationship status → CHA	1.0172	.6318	.1026	1.6101	.1090	[−.2288, 2.2633]
	*c*	SAT → CHA	−.0862	.0434	−.1382	−1.9877	.0482	[−.1718, −.0007]
FIN	*b*_1_	DEP → FIN	−.0229	.0653	−.0295	−.3507	.7262	[−.1516, .1058]
	*b*_2_	FAN → FIN	.1080	.0577	.1427	1.8726	.0626	[−.0057, .2217]
	*b*_3_	PRO → FIN	.1820	.0382	.3862	4.7694	< .001	[.1067, .2572]
	*c′*	SAT → FIN	−.1718	.0480	−.2176	−3.5807	< .001	[−.2664, −.0772]
	−	Age → FIN	−.1622	.0499	−.1898	−3.2524	.0013	[−.2605, −.0638]
	−	Relationship status → FIN	−.7800	.7528	−.0622	−1.0362	.3014	[−2.2647, .7046]
	*c*	SAT → FIN	−.1910	.0522	−.2419	−3.6566	< .001	[−.2940, −.0880]
WOR	*b*_1_	DEP → WOR	−.0840	.0614	−.1250	−1.3699	.1723	[−.2050, .0370]
	*b*_2_	FAN → WOR	.1818	.0542	.2992	3.3538	.0010	[.0749, .2888]
	*b*_3_	PRO → WOR	.0457	.0359	.1207	1.2736	.2043	[−.0251, .1164]
	*c′*	SAT → WOR	−.0205	.0451	−.0322	−.4534	.6507	[−.1094, .0685]
	−	Age → WOR	−.0874	.0451	−.1273	−1.8635	.0639	[−.1798, .0051]
	−	Relationship status → WOR	.1205	.7078	.0120	.1702	.8650	[−1.2754, 1.5164]
	*c*	SAT → WOR	−.0385	.0449	−.0607	−0.8582	.3918	[−.1270, .0500]
MPP	*b*_1_	DEP → MPP	−.0910	.2519	−.0307	−.3610	.7185	[−.5878, .4059]
	*b*_2_	FAN → MPP	.6713	.2226	.2325	3.0157	.0029	[.2323, 1.1104]
	*b*_3_	PRO → MPP	.6217	.1473	.3457	4.2211	< .001	[.3312, .9122]
	*c′*	SAT → MPP	−.2438	.1852	−.0809	−1.3166	.1895	[−.6091, .1214]
	−	Age → MPP	−.6789	.1925	−.2082	−3.5273	< .001	[−1.0585, −.2993]
	−	Relationship status → MPP	−2.3025	2.9061	−.0481	−.7923	.4292	[−8.0341, 3.4291]
	*c*	SAT → MPP	−.3652	.2042	−.1212	−1.7885	.0752	[−.7678, .0375]

*Note*. SAT–life satisfaction; DEP–depressive symptoms; FAN–future anxiety; PRO–procrastination; UNC–feeling of uncertainty and incompetence; SFO–self-focus; BUR–parenthood as a burden; CHA–fear of change; FIN–financial security concern; WOR–worry about a child’s future; MPP–total score for motives for postponing parenthood; *B*–unstandardized regression coefficient; *SE*–standard error; *β*–standardized regression coefficient; CI–confidence interval. Age and relationship status (0 = not currently in a relationship, 1 = currently in a relationship) were included as covariates. Paths *b*_1_–*b*_3_ represent adjusted associations of the intermediate variables with each outcome; *c′* represents the direct association of life satisfaction with the outcome, and c the total association adjusted for the covariates.

Table 5 shows that all full outcome equations were statistically significant, with explained variance differing across motives for postponing parenthood. The highest values were observed for financial security concern and the overall motives score, and the lowest for worry about a child’s future. The covariate-adjusted total-association models generally explained less variance and were statistically significant for all outcomes except worry about a child’s future. Among these models, financial security concern again showed the highest proportion of explained variance.

**Table 5:** Model fit for the full outcome equations and covariate-adjusted total-association models of motives for postponing parenthood (*N* = 201).

Outcome	Full outcome equation *R*²	*F*(df_1_, df_2_)	*p*	Total-association model *R*²	*F*(df_1_, df_2_)	*p*
UNC	.2810	12.6364 (6, 194)	< .001	.0544	3.7743 (3, 197)	.012
SFO	.1938	7.7737 (6, 194)	< .001	.1303	9.8382 (3, 197)	< .001
BUR	.1609	6.2018 (6, 194)	< .001	.0645	4.5248 (3, 197)	.004
CHA	.2763	12.3436 (6, 194)	< .001	.0658	4.6259 (3, 197)	.004
FIN	.3585	18.0686 (6, 194)	< .001	.1547	12.0189 (3, 197)	< .001
WOR	.1213	4.4625 (6, 194)	< .001	.0336	2.2815 (3, 197)	.080
MPP	.3437	16.9365 (6, 194)	< .001	.1134	8.4010 (3, 197)	< .001

*Note*. UNC–uncertainty and incompetence; SFO–self-focus; BUR–parenthood as a burden; CHA–fear of change; FIN–financial security concern; WOR–worry about a child’s future; MPP–overall score for motives for postponing parenthood. Full outcome equations included life satisfaction, depressive symptoms, future anxiety, procrastination, age, and relationship status. Total-association models included life satisfaction, age, and relationship status.

Table 6 presents the total and specific indirect associations between life satisfaction and motives for postponing parenthood, adjusted for age and relationship status. The total indirect association was not supported for any outcome, whereas several specific indirect associations emerged and varied across motives. For uncertainty and incompetence, indirect associations were supported through procrastination, through depressive symptoms and procrastination, and through the full sequence of depressive symptoms, future anxiety, and procrastination. No specific indirect associations were supported for self-focus. For parenthood as a burden and worry about a child’s future, only the sequence through depressive symptoms and future anxiety was supported. Fear of change showed indirect associations through procrastination, depressive symptoms and future anxiety, depressive symptoms and procrastination, and the full sequential sequence. Financial security concern showed the same pattern except for the sequence through depressive symptoms and future anxiety. The overall motives score showed a pattern similar to fear of change. The specific sequential indirect association specified in H6: life satisfaction → depressive symptoms → future anxiety → procrastination → outcome – was supported for uncertainty and incompetence, fear of change, financial security concern, and the total MSMPP-18 score, with 95% bootstrap confidence intervals excluding zero. It was not supported for self-focus, parenthood as a burden, or worry about a child’s future. Thus, H6 received partial support.

**Table 6:** Total and specific indirect associations of life satisfaction with motives for postponing parenthood in model adjusted for age and relationship status (*N* = 201).

Outcome	Effect	Indirect pathway	*B*	BootSE	BootLLCI	BootULCI
UNC	Total	All indirect pathways	−.0269	.0353	−.0976	.0432
	Ind1	SAT → DEP → UNC	−.0082	.0160	−.0455	.0202
	Ind2	SAT → FAN → UNC	−.0056	.0109	−.0358	.0061
	Ind3	SAT → PRO → UNC	.0396	.0192	.0064	.0819
	Ind4	SAT → DEP → FAN → UNC	−.0102	.0091	−.0297	.0072
	Ind5	SAT → DEP → PRO → UNC	−.0295	.0161	−.0660	−.0032
	Ind6	SAT → FAN → PRO → UNC	−.0046	.0063	−.0195	.0064
	Ind7	SAT → DEP → FAN → PRO → UNC	−.0083	.0054	−.0214	−.0007
SFO	Total	All indirect pathways	−.0200	.0235	−.0654	.0277
	Ind1	SAT → DEP → SFO	−.0228	.0177	−.0618	.0073
	Ind2	SAT → FAN → SFO	.0013	.0070	−.0077	.0207
	Ind3	SAT → PRO → SFO	.0148	.0129	−.0046	.0454
	Ind4	SAT → DEP → FAN → SFO	.0024	.0079	−.0145	.0187
	Ind5	SAT → DEP → PRO → SFO	−.0110	.0097	−.0336	.0039
	Ind6	SAT → FAN → PRO → SFO	−.0017	.0031	−.0095	.0028
	Ind7	SAT → DEP → FAN → PRO → SFO	−.0031	.0030	−.0109	.0011
BUR	Total	All indirect pathways	−.0090	.0212	−.0538	.0311
	Ind1	SAT → DEP → BUR	.0176	.0149	−.0043	.0534
	Ind2	SAT → FAN → BUR	−.0090	.0127	−.0387	.0121
	Ind3	SAT → PRO → BUR	.0175	.0111	−.0003	.0430
	Ind4	SAT → DEP → FAN → BUR	−.0164	.0094	−.0373	−.0011
	Ind5	SAT → DEP → PRO → BUR	−.0130	.0095	−.0360	.0005
	Ind6	SAT → FAN → PRO → BUR	−.0020	.0033	−.0109	.0026
	Ind7	SAT → DEP → FAN → PRO → BUR	−.0037	.0029	−.0110	.0001
CHA	Total	All indirect pathways	−.0281	.0317	−.0922	.0332
	Ind1	SAT → DEP → CHA	.0100	.0141	−.0129	.0442
	Ind2	SAT → FAN → CHA	−.0129	.0171	−.0514	.0164
	Ind3	SAT → PRO → CHA	.0256	.0134	.0031	.0536
	Ind4	SAT → DEP → FAN → CHA	−.0234	.0129	−.0523	−.0021
	Ind5	SAT → DEP → PRO → CHA	−.0191	.0116	−.0457	−.0010
	Ind6	SAT → FAN → PRO → CHA	−.0030	.0044	−.0136	.0043
	Ind7	SAT → DEP → FAN → PRO → CHA	−.0054	.0039	−.0148	−.0003
FIN	Total	All indirect pathways	−.0192	.0355	−.0929	.0475
	Ind1	SAT → DEP → FIN	.0049	.0160	−.0265	.0397
	Ind2	SAT → FAN → FIN	−.0074	.0129	−.0439	.0080
	Ind3	SAT → PRO → FIN	.0450	.0196	.0075	.0843
	Ind4	SAT → DEP → FAN → FIN	−.0135	.0099	−.0347	.0037
	Ind5	SAT → DEP → PRO → FIN	−.0335	.0172	−.0719	−.0050
	Ind6	SAT → FAN → PRO → FIN	−.0052	.0070	−.0218	.0067
	Ind7	SAT → DEP → FAN → PRO → FIN	−.0095	.0056	−.0229	−.0012
WOR	Total	All indirect pathways	−.0181	.0265	−.0759	.0289
	Ind1	SAT → DEP → WOR	.0179	.0189	−.0129	.0617
	Ind2	SAT → FAN → WOR	−.0125	.0187	−.0600	.0128
	Ind3	SAT → PRO → WOR	.0113	.0124	−.0125	.0376
	Ind4	SAT → DEP → FAN → WOR	−.0227	.0135	−.0538	−.0016
	Ind5	SAT → DEP → PRO → WOR	−.0084	.0103	−.0322	.0094
	Ind6	SAT → FAN → PRO → WOR	−.0013	.0027	−.0079	.0033
	Ind7	SAT → DEP → FAN → PRO → WOR	−.0024	.0030	−.0092	.0028
MPP	Total	All indirect pathways	−.1213	.1491	−.4278	.1629
	Ind1	SAT → DEP → MPP	.0194	.0620	−.0943	.1633
	Ind2	SAT → FAN → MPP	−.0460	.0663	−.2103	.0524
	Ind3	SAT → PRO → MPP	.1538	.0724	.0219	.3068
	Ind4	SAT → DEP → FAN → MPP	−.0837	.0470	−.1893	−.0080
	Ind5	SAT → DEP → PRO → MPP	−.1146	.0632	−.2574	−.0113
	Ind6	SAT → FAN → PRO → MPP	−.0178	.0241	−.0755	.0238
	Ind7	SAT → DEP → FAN → PRO → MPP	−.0324	.0205	−.0818	−.0027

*Note.* SAT–life satisfaction; DEP–depressive symptoms; FAN–future anxiety; PRO–procrastination; UNC–uncertainty and incompetence; SFO–self-focus; BUR–parenthood as a burden; CHA–fear of change; FIN–financial security concern; WOR–worry about a child’s future; MPP–overall score for motives for postponing parenthood. Age and relationship status were included as covariates. Indirect associations were estimated using 5,000 bootstrap samples. BootSE–bootstrap standard error; BootLLCI and BootULCI–lower and upper limits of the 95% percentile bootstrap confidence interval. Indirect associations were considered supported when the 95% bootstrap CI excluded zero.

Sensitivity analyses were conducted by re-estimating the seven sequential indirect-association models after excluding the three potential multivariate outliers. The sequential indirect association involving depressive symptoms, future anxiety, and procrastination was sensitive to their exclusion. In the full sample, the 95% bootstrap CI excluded zero for four outcomes: uncertainty and incompetence, fear of change, financial security concern, and the total MSMPP-18 score. After exclusion of the three cases, this association remained supported only for uncertainty and incompetence (effect = −0.0065, BootSE = 0.0050, 95% bootstrap CI [−0.0192, −0.0002]). The CIs included zero for fear of change (effect = −0.0041, BootSE = 0.0036, 95% bootstrap CI [−0.0132, 0.0001]), financial security concern (effect = −0.0074, BootSE = 0.0053, 95% bootstrap CI [−0.0206, 0.0000]), and the total MSMPP-18 score (effect = −0.0255, BootSE = 0.0199, 95% bootstrap CI [−0.0767, 0.0002]).

A post hoc Monte Carlo sensitivity analysis indicated substantial variation in estimated power across outcomes, ranging from .188 to .827. Power exceeded .80 for uncertainty and incompetence (.812), financial security concern (.827), and the overall MSMPP-18 score (.808), but was lower for fear of change (.722), parenthood as a burden (.494), self-focus (.267), and worry about a child’s future (.188).

## 4. Discussion

The present study examined a theoretically ordered cross-sectional model linking life satisfaction with motives for postponing parenthood through depressive symptoms, future anxiety, and procrastination. Overall, the findings supported several components of the proposed model, although the sequential indirect-association pattern emerged only for certain motives, suggesting that different reasons for postponement may reflect partly distinct psychological processes.

The findings provided partial support for H1. Life satisfaction was negatively associated with uncertainty and incompetence, fear of change, financial security concern, and the overall motives score, but not with self-focus, parenthood as a burden, or worry about a child’s future. This pattern is broadly consistent with previous research linking subjective well-being with reproductive intentions and behavior [89] and with evidence that economic insecurity and broader life circumstances are relevant to reproductive decision-making [90,91]. The lack of negative associations with the remaining motives indicates that they may reflect factors other than lower well-being. The positive adjusted association between life satisfaction and self-focus further suggests that prioritizing personal goals before parenthood may be compatible with higher well-being.

Hypotheses H2 and H3 were supported, showing the expected associations between life satisfaction and depressive symptoms and between depressive symptoms and future anxiety. Higher life satisfaction was associated with fewer depressive symptoms, consistent with longitudinal evidence linking subjective well-being and depressive symptomatology [92]. Depressive symptoms were, in turn, positively associated with future anxiety, in line with cognitive accounts emphasizing hopelessness, reduced positive expectations, and negative representations of future events [45,93–95]. Notably, the association between life satisfaction and future anxiety was no longer significant after depressive symptoms and covariates were included, suggesting that this relationship may largely overlap with depressive symptomatology.

Consistent with H4, future anxiety was positively associated with procrastination, in line with previous research linking anxiety, uncertainty, and negative future orientation with procrastinatory tendencies [53,96–98]. Similar findings were reported by Przepiórka et al. [53], who found that higher general procrastination was associated with greater future anxiety among university students. This association may reflect the emotion-regulation function of procrastination, whereby engagement with emotionally aversive or uncertain situations is delayed to obtain short-term relief [63, 64]. Because procrastination was assessed as a generalized dispositional tendency rather than as parenthood-specific behavior, this finding is best understood as evidence of a link between future anxiety and general procrastinatory tendencies.

Hypothesis H5 was also supported. Procrastination was positively correlated with all six motives for postponing parenthood and with the overall motives score. In the adjusted models, however, unique positive associations remained for uncertainty and incompetence, parenthood as a burden, fear of change, financial security concern, and the overall score, but not for self-focus or worry about a child’s future. This pattern is consistent with theoretical accounts of procrastination emphasizing avoidance of aversive, uncertain, or demanding situations and difficulties in self-regulation [63, 64]. Motives involving perceived unreadiness, anticipated burden, change, and financial insecurity may make the transition to parenthood appear less manageable and therefore be more closely related to procrastinatory tendencies. By contrast, self-focus and concerns about a child’s future may be more strongly rooted in lifestyle priorities, personal values, or broader societal considerations.

The central hypothesis, H6, received partial support. The sequential indirect association through depressive symptoms, future anxiety, and procrastination was supported for uncertainty and incompetence, fear of change, financial security concern, and the overall motives score, but not for self-focus, parenthood as a burden, or worry about a child’s future. Importantly, the sequential analysis adds to the individual associations examined in H1–H5 by showing that only some postponement motives were linked to life satisfaction through the full proposed psychological sequence. The associations between lower life satisfaction and motives concerning readiness, anticipated change, and financial security were consistent with a broader pattern involving depressive symptoms, greater future anxiety, and procrastinatory tendencies. The indirect-association findings therefore provide a more differentiated account of postponement motives than would be obtained from considering the individual associations alone.

This outcome-specific pattern is broadly consistent with previous research suggesting that motives for postponing parenthood differ in their psychological correlates. Anxiety and procrastination have been linked to perceived parental incompetence, fear of change, and financial concerns, whereas self-focus appears less consistently related to anxiety and procrastination [3, 4]. The supported sequential associations may therefore identify motives that are especially sensitive to perceived personal unreadiness, anticipated uncertainty, and insufficient resources. In contrast, self-focus may be more closely related to self-development and lifestyle priorities, while worry about a child’s future may reflect broader societal and environmental concerns. The absence of a sequential association for parenthood as a burden, despite its independent association with procrastination, further suggests that this motive may be related to procrastinatory tendencies through processes not captured by the full proposed sequence. Differences in explained variance across the outcome models further support this interpretation. The full models explained the greatest proportion of variance in financial security concern and the overall motives score, whereas substantially less variance was explained in worry about a child’s future. This finding further supports the heterogeneity of postponement motives and highlights the value of examining specific motives rather than relying solely on a global postponement score.

The indirect-association results revealed a more complex configuration than a simple linear sequence. Although previous research generally indicates a negative association between procrastination and life satisfaction [99,100], these variables were not significantly correlated in the present study. Similar nonsignificant associations have been reported in some samples [101], suggesting that this relationship may vary across populations. Notably, a positive association emerged after depressive symptoms, future anxiety, age, and relationship status were controlled. This change in direction may reflect a suppression-like effect, whereby controlling for related variables reveals an association that is not apparent at the bivariate level [102]. In addition, several indirect associations involving depressive symptoms and future anxiety were negative, which may help explain why the total indirect association was not significant for any outcome despite several supported specific indirect associations. Overall, the proposed sequence did not operate uniformly across all associations and postponement motives, and some relationships became apparent only after other psychological variables were taken into account. Given the cross-sectional design, the observed sequential indirect associations should be interpreted as consistent with the theoretically proposed ordering rather than as evidence of temporal or causal mediation. Reciprocal, parallel, and alternative configurations remain plausible and should be examined in longitudinal research.

Finally, the motive-specific results warrant caution because the six MSMPP-18 subscales were correlated and overlapped with the overall score. The total MSMPP-18 score was therefore treated as the primary outcome, whereas the subscale analyses were used to characterize heterogeneity across motives. Sensitivity analyses also indicated that some outcome-specific sequential associations were less robust than the broader regression findings. Accordingly, the strongest conclusions concern the overall pattern and differentiation across motives rather than isolated subscale effects.

### 4.1. Limitations and future directions

Several limitations should be considered when interpreting the findings. First, the cross-sectional design precludes conclusions about temporal ordering or causality among the study variables. Although the ordering of life satisfaction, depressive symptoms, future anxiety, and procrastination was specified a priori on theoretical grounds, alternative directional, reciprocal, or competing models cannot be ruled out. Longitudinal research is needed to determine whether the proposed associations unfold over time and whether these psychological characteristics prospectively predict changes in motives for postponing parenthood.

Second, the convenience sampling procedure limits the generalizability of the findings. The results should therefore be interpreted primarily in relation to childless Polish women within the age range represented in the present sample and should not be assumed to generalize to men, parents, women outside this age range, or women from other national and cultural contexts. Replication in larger, more representative, and culturally diverse samples would help establish the robustness of the observed associations.

Third, the study assessed self-reported motives for postponing parenthood rather than actual reproductive behavior. We did not determine whether participants subsequently delayed childbearing, changed their fertility intentions, initiated attempts to conceive, or experienced specific fertility outcomes. The sample may also have been heterogeneous with respect to reproductive intentions and experiences, as we did not distinguish between women who intended to have children, were undecided, were voluntarily childfree, were trying to conceive, or had experienced infertility, pregnancy loss, or medical contraindications to pregnancy. These factors may influence how motives for postponing parenthood are understood and reported. All variables were assessed using self-report measures at a single time point, which may have increased common method variance. In addition, employment status, income, and perceived economic situation were not assessed. Because financial security concern was one of the specific motives for postponement, unmeasured socioeconomic factors may have contributed to the observed associations with this outcome.

The a priori power analysis was based on a bivariate correlation and did not specifically estimate power for the sequential indirect-association models. The post hoc Monte Carlo analysis indicated substantial variation in sensitivity across outcomes, suggesting that small outcome-specific indirect associations may have been difficult to detect. Moreover, because multiple correlated outcome-specific models were examined, the subscale findings should be interpreted cautiously and replicated in independent samples.

Finally, sensitivity analyses indicated that the sequential indirect association was not fully robust to the exclusion of three potential multivariate outliers. Although the broader regression findings remained substantively unchanged, some outcome-specific sequential associations appear less robust and should therefore be interpreted with caution.

### 4.2. Implications

The present findings suggest that psychological characteristics may be relevant to understanding some self-reported motives for postponing parenthood, but that their relevance differs across specific reasons for postponement. Recognizing this heterogeneity may be useful for professionals working with individuals who experience uncertainty or distress in relation to reproductive decisions. Rather than treating postponement itself as problematic, psychological support may focus on helping individuals clarify their reproductive goals, distinguish deliberate and values-based postponement from reproductive decisions accompanied by distress, anxiety, or avoidance, and develop adaptive strategies for managing difficult emotions when these are personally relevant. Reproductive decision-making should also be considered in the context of relational, economic, occupational, and social circumstances. Because the present observational study did not examine actual reproductive behavior or test any intervention, stronger clinical, educational, or policy recommendations would be premature.

## 5. Conclusions

The present study identified distinct associations between life satisfaction, depressive symptoms, future anxiety, procrastination, and self-reported motives for postponing parenthood among childless Polish women. The hypothesized sequential indirect association through depressive symptoms, future anxiety, and procrastination received partial and outcome-specific support, indicating that the proposed pattern was more evident for some motives than for others.

The absence of supported total indirect associations and the presence of competing specific indirect associations suggest that the relationships among these variables are more complex than a single unidirectional sequence. Taken together, the findings support viewing motives for postponing parenthood as psychologically heterogeneous and caution against reducing reproductive decision-making to a single psychological explanation. They may also contribute to a better understanding of the psychological characteristics associated with uncertainty and ambivalence surrounding decisions about parenthood. Given the cross-sectional design, these findings should be interpreted as evidence of associations rather than as evidence of a causal psychological mechanism.
